# Histopathologic study of extraocular muscles in thyroid-associated ophthalmopathy coexisting with ocular myasthenia gravis: a case report

**DOI:** 10.1186/s12886-020-01431-y

**Published:** 2020-04-22

**Authors:** Ruiqi Ma, Yun Cheng, Lu Gan, Xiaoting Zhou, Jiang Qian

**Affiliations:** 1 Department of Ophthalmology, Fudan Eye & ENT Hospital, Shanghai, China; 2 NHC Key Laboratory of Myopia, Fudan University, Shanghai, China; 3 Laboratory of Myopia, Chinese Academy of Medical Sciences, Shanghai, China

**Keywords:** Thyroid-associated ophthalmopathy, Ocular myasthenia gravis, Histopathology, Extraocular muscles

## Abstract

**Background:**

Coexistence of thyroid-associated ophthalmopathy (TAO) and ocular myasthenia gravis (OMG) is very rare. Little is known about the orbital histopathology associated with this condition. The authors reported a case of TAO coexisting with OMG and explored the histopathologic changes in extraocular muscles.

**Case presentation:**

A 32-year-old man complaint of bilateral proptosis for 2 years. The patient was documented with a history of OMG and was treated with blepharoplasty to correct ptosis 3 years prior to presentation. Physical examination revealed right upper eyelid retraction resulting from the eyelid surgery. Computed tomographic scan demonstrated bilateral enlargement of the extraocular muscles. Thyroid function test confirmed hyperthyroid status. The patient was diagnosed with TAO (clinical activity score = 2/7) coexisting with OMG. Orbital decompression surgery reduced proptosis but resulted in new onset of left upper eyelid retraction because of the increased motor impulses to sustain eyelid elevation. Extraocular muscles were sampled during surgery and subjected to histopathologic stain. The stain results were analyzed against samples from age-, gender- matched TAO and control (non-TAO non-OMG) subjects. The measurement of myofiber size and glycosaminoglycan/collagen-occupied area was repeated in 3 randomly chosen fields of each slide. The variation of myofiber size was larger in the TAO + OMG (289.9 ± 142.5 μm^2^) samples than the TAO (544.1 ± 160.6 μm^2^) and control (157.0 ± 47.7 μm^2^) samples. Glycosaminoglycan was more abundant in the TAO + OMG (48.8 ± 12.2%) samples than the TAO (28.4 ± 3.6%) and control (3.3 ± 0.8%) samples. Collagen fibers accumulated in the TAO (60.5 ± 6.4%) samples but not in the TAO + OMG (36.1 ± 4.3%) and control (33.9 ± 2.7%) samples. Typical OMG changes were observed in the TAO + OMG samples but not in the TAO and control samples. These changes included central nuclei, aggregation of mitochondria and fiber type grouping. The histopathologic findings of TAO + OMG were summarized as inhomogeneously enlarged muscle fibers and predominantly endomysial accumulation of glycosaminoglycan.

**Conclusion:**

This study highlights the possibility of TAO coexisting with OMG and demonstrates the histopathologic features in this rare condition.

## Background

Thyroid-associated ophthalmopathy (TAO), an autoimmune disorder of orbital tissues, commonly coexists with autoimmune thyroid diseases such as Graves’ disease [1]. Besides thyroid dysfunction, 18.9% of the TAO patients were reported in a large case series with a second autoimmune disease, among which vitiligo (17.4%), chronic autoimmune gastritis (14.4%) and rheumatoid arthritis (12.8%) were most frequently identified [2]. Myasthenia gravis (MG) was also reported in this case series, but its prevalence was relatively low and was calculated as 0.1% in the TAO cases. MG is an autoimmune condition characterized by the symptom of muscle weakness which results from autoantibodies to neuromuscular junctions. Patients with symptoms confined to ocular muscles are classified as ocular MG (OMG) [3]. Approximately 15.0% of MG patients have a concurrent autoimmune disease, but co-occurrence of TAO and OMG is very rare [4]. As far as we know, only a few cases were reported in previous literature, and little is known about the histopathologic features underlying this rare condition [5]. Herein, the authors describe a case of TAO coexisting with OMG and compare the histopathologic findings in this patient with 3 TAO and 3 control (non-TAO non-OMG) subjects (matched by age and gender). This study was conducted in compliance with the Declaration of Helsinki, and patient consent was obtained for publishing identifiable photographs.

## Case presentation

**Fig. 1:**
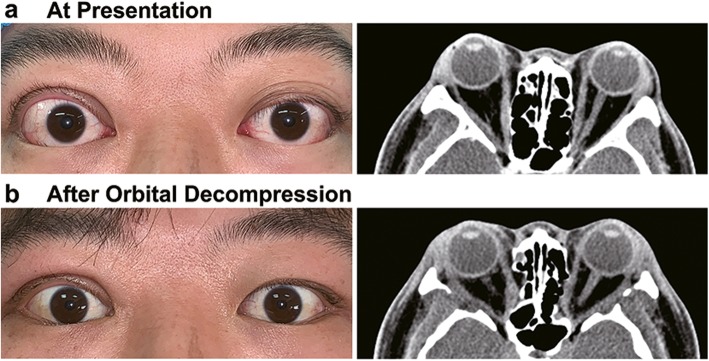
Case presentation (TAO with OMG). **a** A 32-year-old male complaint of bilateral proptosis for 2 years. Physical examination revealed slight ptosis of the left upper eyelid and mild retraction of the right upper eyelid. The patient reported a medical history of OMG, and the unnatural crease of the right eyelid resulted from previous blepharoplasty to correct ptosis. The CT scan showed bilateral enlargement of the medial rectus muscles. **b** Orbital decompression partially relieved the right upper eyelid retraction but resulted in new onset of left upper eyelid retraction due to increased motor impulses to sustain eyelid elevation. The CT scan showed balanced two-wall decompression in both orbits

A 32-year-old man presented to a tertiary eye center with a 2-year progression of bilateral proptosis. Physical examination revealed prominent proptosis, mild conjunctival redness, inflamed plica and lower eyelid retraction in both eyes. The unnatural crease of the right upper eyelid resulted from an eyelid surgery 3 years prior to presentation (Fig. 1a, left). Before the eyelid surgery, he was documented with a 1-year history of right eye ptosis which worsened upon sustained upward gaze. The patient was suspected with OMG and underwent relative examinations. Both the neostigmine test and the ice-pack test reversed ptosis. The neurophysiological tests revealed no involvement of non-ocular muscles. Computed tomographic (CT) scan demonstrated thymus enlargement. Although serologic tests detected no antibodies against acetylcholine receptors, the other clinical findings secured the diagnosis of OMG. The patient underwent total thymectomy, and the histopathologic diagnosis was thymic hyperplasia. After surgery, the ocular symptoms showed no improvement. The patient was then treated with right eye blepharoplasty to “correct ptosis” in an oculoplastic medical facility. The surgery partially relieved the right eye ptosis but resulted in mild drooping of the left eyelid. One year later, the symptom of bilateral proptosis developed.

At presentation, the patient reported no symptoms of diplopia, numbness, orbicularis weakness, or generalized weakness. Extraocular motility showed slight restriction at upgaze in both eyes. Hertel exophthalmometer readings were 23 mm in the right eye and 25 mm in the left eye. CT scan revealed bilateral enlargement of the inferior rectus and inferior oblique, and mild thickening of the medial rectus and superior rectus muscles in both eyes (Fig. 1a, right). His thyroid function was consistent with the laboratory findings of Graves’ disease, showing decreased thyroid-stimulating hormone (TSH) and positive TSH receptor antibody (see Table 1 in Additional file 1 which demonstrates the laboratory test results of this patient). After consultation with endocrinologist, the patient was prescribed with methimazole to rehabilitate thyroid function. In terms of smoking status, the patient was a current smoker with an average of 6 cigarettes per day for 4 years. Based on the medical history and the clinical findings, a diagnosis of TAO coexisting with OMG was established. The clinical activity score (CAS) of TAO was rated as 2 out of 7 based on the congestive conjunctiva and the inflamed caruncle/plica. The CAS suggested inactive stage of TAO, and neither glucocorticoid nor radiotherapy was considered effective in inactive TAO. Once retrieved euthyroid status, the patient underwent balanced two-wall decompression in both orbits to reduce proptosis (Fig. 1b, right). The right eyelid retraction improved after surgery, but the left eye developed new onset of upper eyelid retraction (Fig. 1b, left). This symptom probably resulted from increased motor impulses to sustain eyelid elevation [8].

**Table 1:** Histopathologic findings of extraocular muscles in OMG reported in literature

Light Microscopy	Electron Microsopy
Myofiber atrophy, central nucleus	Myofibrillar loss and disarray, Z-line streaming
Fatty replacement of myofibers, lipid vacuoles	Subsarcolemmal aggregates of swollen mitochondria
Lymphocytic infiltration	Intramyocellular/Intranuclear lipid vacuoles
Sporadic aggregation of mitochondria	Collagen deposition between myofibers
Others: focal thickening of basal lamina, intermyofibrillar deposition of glycogen	Others: pyknotic nucleus, sarcoplasmic reticulum dilatation, autophagic vacuoles containing lipofuscins

OMG, ocular myasthenia gravis

References: Europa TA et al. 2019 [6], Rautenbach RM et al. 2017 [7]

**Fig. 2:**
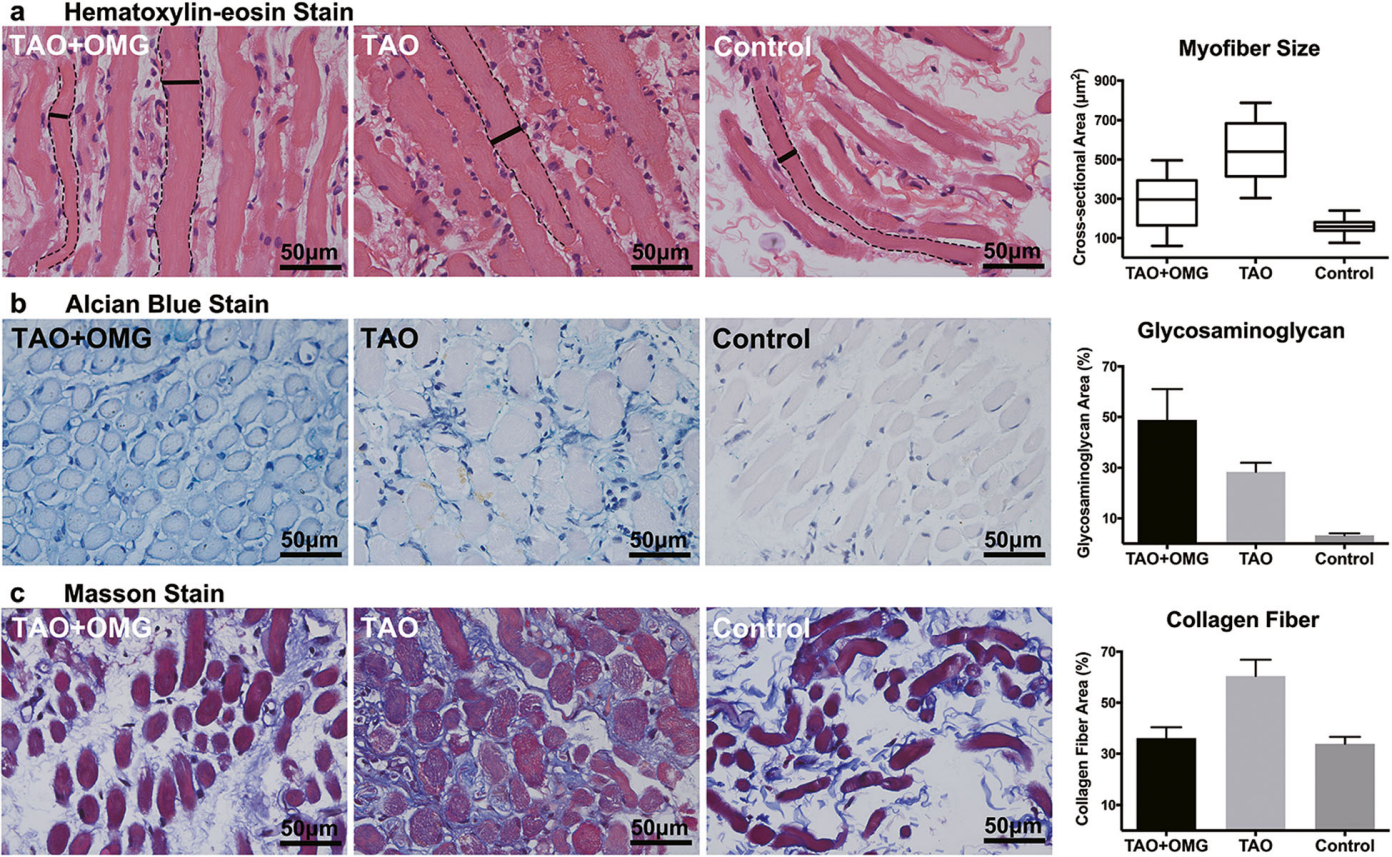
Histopathologic changes in TAO + OMG, TAO and control extraocular muscle samples. **a** Hematoxylin-eosin stain (× 400) showed diffuse inflammation and myofiber enlargement in the TAO + OMG and TAO samples. The cross-sectional area of each myofiber was calculated based on the largest diameter (solid line) perpendicular to the longitudinal axis (dashed line) measured in 3 randomly chosen fields of each slide. The myofiber size was larger in the TAO + OMG (289.9 ± 142.5 μm^2^) and TAO (544.1 ± 160.6 μm^2^) samples than the control samples (157.0 ± 47.7 μm^2^). The variation of myofiber size was smaller in the TAO (± 29.5% around average) and control (± 30.4% around average) samples than the TAO + OMG samples (± 49.2% around average). The bars represent the 95% confidential interval. The box represents the mean and standard deviation. **b** Alcian blue stain (× 400) demonstrated accumulation of glycosaminoglycan (*blue*) in the TAO + OMG and TAO samples. The proportion of glycosaminoglycan was quantified in 3 randomly chosen fields of each slide based on the fraction of positive-stained pixels in the total pixels of the interstitial space. The glycosaminoglycan-occupied area was larger in the TAO + OMG samples (48.8 ± 12.2%) than the TAO (28.4 ± 3.6%) and control (3.3 ± 0.8%) samples. **c** Masson stain (× 400) revealed increased collagen fibers (*blue*) in the TAO samples but not in the TAO + OMG samples. The proportion of collagen fibers in the interstitial space was quantified in 3 randomly chosen fields of each slide based on pixel counting. The collagen-occupied area was larger in the TAO samples (60.5 ± 6.4%) than in the TAO + OMG (36.1 ± 4.3%) and control (33.9 ± 2.7%) samples

**Fig. 3:**
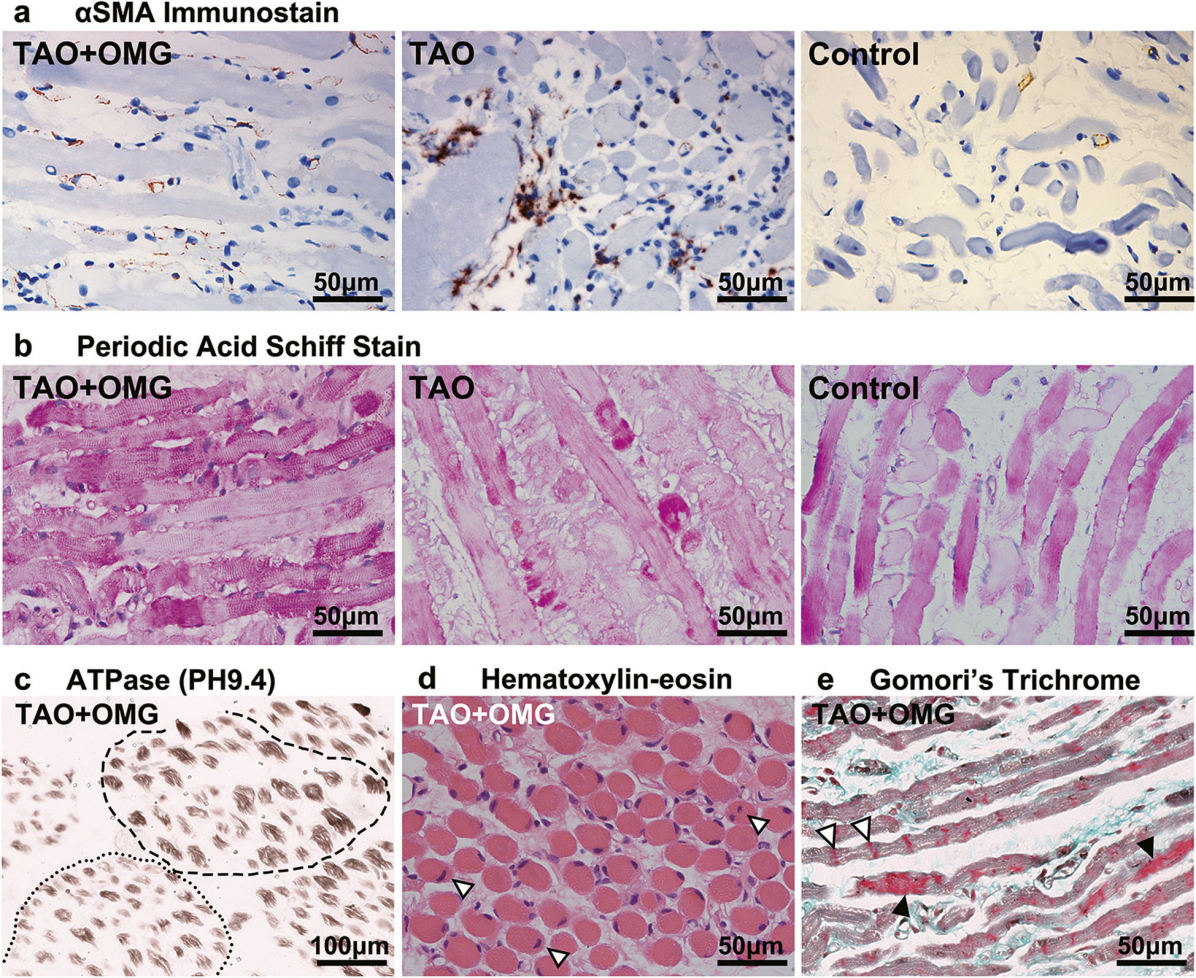
Other histopathologic changes in TAO + OMG, TAO and control extraocular muscle samples. **a** The immunostaining of αSMA (× 400), a potential marker of fibrosis, revealed stronger reactivity (*brown*) in the TAO samples than in the TAO + OMG and control samples. **b** The periodic acid Schiff stain (× 400) demonstrated glycogen (*red*) in the muscle fibers, showing endomysial aggregation of glycogen in the TAO samples and segmental deposition of glycogen in the TAO + OMG samples. **c** Muscle types were distinguished based on the ATPase stain (× 200) of frozen sections, with weak intensity for type I fibers and strong intensity for type II fibers. Same fiber types tended to group together in the TAO + OMG samples, resulting in predominantly type I fibers in some fascicles (*dotted line*) and type II fibers in others (*dashed line*). **d** Central nuclei (*white arrowheads*) were frequently observed in the TAO + OMG samples on hematoxylin-eosin stain (× 400) but not in the TAO or control samples. **e** The Gomori’s trichrome stain (× 400) of frozen sections revealed mitochondrial distribution (*red*) in the TAO + OMG samples, showing both a normal pattern of linear distribution (*white arrowheads*) and an abnormal pattern of focal aggregation (*black arrowheads*)

Small samples of the lateral rectus (2–3 mm length, 1–2 mm diameter) were obtained from both eyes during the decompression surgery. The pathology department provided paraffin blocks of the extraocular muscles obtained from 3 age-, gender- matched TAO subjects and 3 non-TAO non-OMG subjects (see Table 2 and 3 in Additional file 1 which demonstrate the clinical features of the recruited subjects). These samples were subjected to histopathologic stain, and bright-field images were taken with Leica microscope. Data were repeatedly measured in 3 randomly chosen fields of each slide and analyzed with Image J software. Hematoxylin-eosin stain demonstrated diffuse lymphocytic infiltration and myofiber hypertrophy in the TAO + OMG and TAO samples (Fig. 2a). The variation of myofiber size was relatively larger in the TAO + OMG samples compared with the TAO and control samples (Fig. 2a). Alcian blue stain revealed accumulation of glycosaminoglycan (Fig. 2b *blue*) in the TAO + OMG and TAO samples, and the glycosaminoglycan-occupied area was much larger in the TAO + OMG samples than in the TAO samples (Fig. 2b). Masson stain demonstrated accumulation of collagen fibers (Fig. 2c *blue*) in the TAO samples but not in the TAO + OMG samples (Fig. 2c). The αSMA immunostain results were consistent with the Masson results, showing stronger reactivity in the TAO samples than in the TAO + OMG samples (Fig. 3a *brown*). The glycogen in myofibers was revealed by periodic acid Schiff stain (Fig. 3b *red*), showing that glycogen aggregated at the endomysial sites in the TAO samples and segmentally distributed in the control samples. Both features were observed in the TAO + OMG samples, suggesting the pathogenesis of TAO + OMG may differ from TAO. Typical histopathologic findings of OMG were also observed in the TAO + OMG samples, including fiber type grouping (one fiber surrounded by fibers of the same histologic type) on ATPase stain (Fig. 3c, weak for type I fibers and strong for type II fibers), central nuclei on hematoxylin-eosin stain (Fig. 3d, *white arrowheads*), and focal aggregation of mitochondria on Gomori’s trichrome stain (Fig. 3e, *black arrowheads*). The histopathologic features in the TAO + OMG samples were summarized as inhomogeneously enlarged myofibers, remarkable deposition of glycosaminoglycan, and a lack of collagen fiber accumulation.

## Discussion and conclusions

Co-occurrence of TAO and MG is very rare. Based on a retrospective study of 1482 MG cases, only 20 cases (1.3%) were identified with TAO [9]. Several risk factors may contribute to the development of coexisting autoimmune disorders, including early-onset of MG, thymic hyperplasia and previous thymectomy. Early-onset MG is defined as an onset of MG before the age of 50 year-old. This subgroup is associated with HLA-DR3, HLA-B8, and non-HLA genes that are known to influence the immune system [4]. The early-onset MG also has propensity to coexist with thymic hyperplasia which aggravates the development of concurrent autoimmune diseases [9]. Thymectomy is another risk factor for new onset of autoimmune disorders. In a multicenter study of 85 patients who underwent thymectomy, 7 patients (8.2%) developed autoimmune disorders after surgery [10]. As a results, the treatment method for thymic hyperplasia remains controversial. In our study, the patient was an early-onset MG with a medical history of thymectomy due to thymic hyperplasia. The risk factors mentioned above were identified in this case and thus may collectively contribute to the pathogenesis of concurrent TAO and OMG.

Previous studies have unveiled several histopathologic features of extraocular muscles in TAO and in OMG patients. In TAO, the extraocular muscles are characterized by lymphocytic infiltration, myofiber hypertrophy, hyaluronan accumulation, and deposition of collagen fibrils [11]. Our findings in the TAO samples are consistent with these typical changes. In OMG, the extraocular muscles were reported with muscle fiber atrophy and mitochondrial abnormalities (Table 1). These distinctive features are attributed to impaired neuromuscular transmission and neurogenic muscle weakness [6]. Owing to the rarity of concurrent TAO and OMG, little is known about the histopathologic findings of extraocular muscles in this rare condition. Based on our study, both the TAO and OMG features were observed in the TAO + OMG samples. The TAO-related changes included hyaluronan deposition and endomysial aggregation of glycogen, and the coexisting OMG-related changes included central nuclei, focal distribution of mitochondria and fiber type grouping. The typical change in the TAO + OMG samples is larger deviation of myofiber size than the TAO and control samples. This phenomenon probably results from a mutual interaction between TAO-related hypertrophy and MG-related atrophy [7]. Moreover, we noticed several other distinctive features in the TAO + OMG samples, including lack of fibrosis (typical in TAO), absence of fatty replacement (typical in OMG) and focal aggregation of hyaluronan at the endomysial sites. These findings collectively suggest that coexistence of TAO and OMG may follow a distinct pathogenic process rather than an overlapping process of TAO and OMG. Notably, a growing evidence supports the concept that the male TAO patients are more prone to develop active and severe clinical symptoms than the female patients [12]. Previous studies also unveiled a gender bias in co-occurrence of thyroid disease and MG, suggesting that the prevalence in female population was higher than the male population [9, 10]. Since all subjects we recruited were male, it is necessary to address that the data in our study can not represent the real distribution in general population.

To conclude, the authors reported the clinical and histopathologic features of a rare case with co-occurrence of TAO and OMG. The study highlights the possibility of coexisting conditions in TAO and OMG patients and provides evidence of unique pathogenic changes in this rare condition. Further study is necessary to investigate the pathogenesis of coexisting autoimmune disorders.

## Supplementary information

**Additional file 1.** Demonstrates the clinical information of the TAO + OMG, TAO and control subjects.

## Data Availability

The datasets used and/or analyzed during the current study are available from the corresponding author on reasonable request.

## Abbreviations

TAO: Thyroid-associated ophthalmopathy
MG: Myasthenia gravis;
OMG: Ocular myasthenia gravis
CT: Computed tomographic
TSH: Thyroid-stimulating hormone
CAS: Clinical activity score
