# Supplementary material for: Histopathologic study of extraocular muscles in thyroid-associated ophthalmopathy coexisting with ocular myasthenia gravis: a case report
Source: BMC Ophthalmol. 2020 Apr 22;20:166. doi: 10.1186/s12886-020-01431-y (PMC7178726; doi:10.1186/s12886-020-01431-y)
Supplement: Supplementary file 1 — Additional file 1. Demonstrates the clinical information of the TAO + OMG, TAO and control subjects. [file 12886_2020_1431_MOESM1_ESM.docx]

| Table 1. Laboratory test results of the TAO+OMG patient at presentation | | | | |
| --- | --- | --- | --- | --- |
| Test | **Item** | **Result** | **Reference Range** | **Unit** |
| Thyroid Function | Total Triiodothyronine (TT3) | 1.06 | 0.58-1.59 | ng/ml |
|  | Total Tetraiodothyronine (TT4) | 7.15 | 4.87-11.72 | ug/dl |
|  | Free Triiodothyronine (FT3) | 3.82 ↑ | 1.71-3.71 | pg/ml |
|  | Free Tetraiodothyronine (FT4) | 0.98 | 0.70-1.48 | ng/dl |
|  | Thyroid-stimulating Hormone (TSH) | 0.012 ↓ | 0.350-4.940 | uIU/ml |
| Thyroid-associated Autoimmune Antibodies | Thyroid-stimulating Hormone Receptor Antibody (TSHR-Ab) | 8.45 ↑ | 0.00-1.50 | IU/L |
|  | Thyroid Peroxidase Antibody (TPO-Ab) | 0.71 | 0.00-5.61 | IU/ml |
|  | Thyroglobulin Antibody (Tg-Ab) | 0.68 | 0-4.1100 | IU/ml |
| MG-associated Autoimmune Antibodies | Acetylcholine Receptor Antibody (AchR-Ab) | 0.20 | <0.50 | nmol/L |
|  | Muscle-specific Kinase Antibody (MuSK-Ab) | 0.14 | <0.40 | U/ml |
|  | Titin Antibody (TIN-Ab) | 1.16 | <1.99 | nmol/L |
| Erythrocyte Sedimentation Rate | Erythrocyte Sedimentation Rate (ESR) | 4 | ≤15 | mm/h |
| C-Reactive Protein | C-Reactive Protein (CRP) | 1.090 | <3.000 | mg/L |
| TAO, thyroid-associated ophthalmopathy  OMG, ocular myasthenia gravis  MG, myasthenia gravis | | | | |

| Table 2. Clinical information of the recruited TAO subjects | | | | | | | |
| --- | --- | --- | --- | --- | --- | --- | --- |
| No. | **Gender** | **Age** | **Onset of TAO**  **(months)** | **CAS**  **(scale: 0-7)** | **Glucocorticoid/Radiotherapy (within six-months)** | **Onset of Graves’ Disease**  **(months)** | **Thyroid Condition**  **(at presentation)** |
| TAO-1 | Male | 35 | 8 | 2 | Orbital Radiotherapy (18Gy per eye) | 8 | Euthyroid |
| TAO-2 | Male | 43 | 6 | 4 | Intravenous Methylprednisolone (3.5g) | 12 | Euthyroid |
| TAO-3 | Male | 48 | 4 | 4 | Intravenous Methylprednisolone (3.0g) | 6 | Euthyroid |
| TAO, thyroid-associated ophthalmopathy  CAS, clinical activity score. The CAS is rated on a scale from 0 to 7 with one point for each following item: eyelid swelling, eyelid erythema, conjunctival redness, chemosis, caruncle or plical inflammation, spontaneous orbital pain, and gaze evoked orbital pain. | | | | | | | |

| Table 3. Clinical information of the recruited control subjects | | | | |
| --- | --- | --- | --- | --- |
| No. | **Gender** | **Age** | **Diagnosis** | **Surgery** |
| Control-1 | Male | 34 | Cavernous Hemangioma | Removal of Orbital Mass |
| Control-2 | Male | 44 | Orbital Fracture | Orbital Plasty |
| Control-3 | Male | 48 | Cavernous Hemangioma | Removal of Orbital Mass |
